# Solitary osteochondroma of the cervical spine presenting as recurrent torticollis

**DOI:** 10.11604/pamj.2014.17.271.3977

**Published:** 2014-04-13

**Authors:** Ali Akhaddar, Mohamed Boucetta

**Affiliations:** 1 Department of Neurosurgery, Avicenne Military Hospital, Marrakech, Morocco; 2 University of Mohammed V Souissi, Rabat, Morocco

**Keywords:** Solitary osteochondroma, torticollis

## Image in medicine

A 48-year-old man was referred to our unit for assessment of recurring episodes of painful torticollis. Family and past histories were unremarkable. There was no traumatic antecedent. During the previous three years he had experienced several episodes of torticollis and painful cervical movements without radiculopathy. His neurological examination was normal, except for a head tilt, decrease range of cervical motion and local tenderness on the right lateral side of the neck. Cervical spine radiographs showed a cervical scoliosis with right unilateral C5-C6 facet joint hypertrophy (A). Cervical computed tomography-scan and magnetic resonance imaging demonstrated a small bone regular tumor in the right C6 articular process and important amyotrophia of the neck musculature on the right side without nerve root or spinal cord compression (B and C). A posterior cervical approach was performed and the mass was completely removed without facet joint sacrifice. At surgery, the tumor appeared well-circumscribed, firm, and calcified with a cartilaginous-cap like appearance (D). Histological features were consistent with benign osteochodroma. The patient was discharged home pain free and referred for physiotherapy care with a good outcome. Osteochondroma is the most common benign tumor of bone (especially long bones), but the spine is rarely involved and usually indicates a hereditary cause such as osteochondromatosis (hereditary multiple exostosis). As seen in our case, this lesion is slow growing, and therefore significant spinal deformation can occur before the symptoms are recognized.

**Figure 1:**
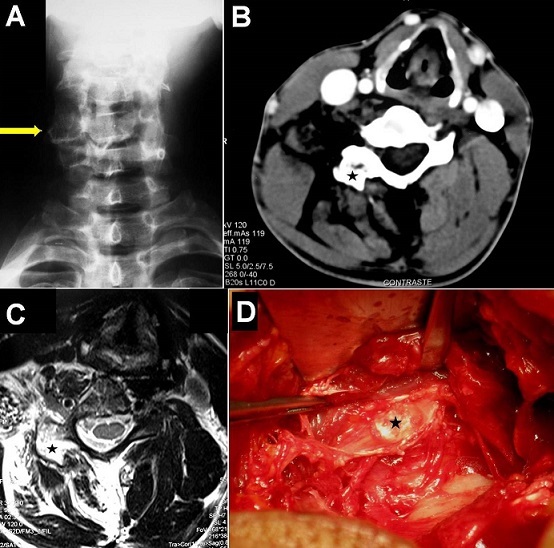
(A) Antero-posterior cervical radiograph showing the scoliosis with right unilateral C5-C6 facet joint hypertrophy (arrow). (B and C) Axial CT-scan and T2-weighted MR imaging demonstrating the osseous well-circumscribed tumor (star) in the right C6 articular process and diffuse amyotrophia of the neck musculature on the right side without nerve root or spinal cord compression. (D) Intraoperative photograph of the calcified cartilaginous tumor (star) before removal

